# Systematic review of studies evaluating the broader economic impact of vaccination in low and middle income countries

**DOI:** 10.1186/1471-2458-12-878

**Published:** 2012-10-16

**Authors:** Rohan Deogaonkar, Raymond Hutubessy, Inge van der Putten, Silvia Evers, Mark Jit

**Affiliations:** 1 Health Economics Unit, University of Birmingham, Birmingham, UK; 2 Initiative for Vaccine Research, World Health Organization, Geneva, Switzerland; 3 Department of Health Services Research, Maastricht University, Maastricht, The Netherlands; 4 Modelling and Economics Unit, Health Protection Agency, London, UK; 5 Department of Infectious Disease Epidemiology, London School of Hygiene and Tropical Medicine, London, UK

**Keywords:** Economic evaluations, Cost-effectiveness analysis, Externalities, Vaccines, Immunization, Low and middle income countries, Developing countries

## Abstract

**Background:**

Most health economic evaluations of childhood vaccination only capture the health and short-term economic benefits. Measuring broader, long-term effects of vaccination on productivity and externalities could provide a more complete picture of the value of vaccines.

**Method:**

MEDLINE, EconLit and NHS-EED databases were searched for articles published between January 1990 and July 2011, which captured broader economic benefits of vaccines in low and middle income countries. Studies were included if they captured at least one of the following categories on broader economic impact: outcome-related productivity gains, behaviour-related productivity gains, ecological externalities, equity gains, financial sustainability gains or macroeconomic benefits.

**Results:**

Twenty-six relevant studies were found, including observational studies, economic models and contingent valuation studies. Of the identified broader impacts, outcome-related productivity gains and ecological externalities were most commonly accounted for. No studies captured behaviour-related productivity gains or macroeconomic effects. There was some evidence to show that vaccinated children 8–14 years of age benefit from increased cognitive ability. Productivity loss due to morbidity and mortality was generally measured using the human capital approach. When included, herd immunity effects were functions of coverage rates or based on reduction in disease outcomes. External effects of vaccines were observed in terms of equitable health outcomes and contribution towards synergistic and financially sustainable healthcare programs.

**Conclusion:**

Despite substantial variation in the methods of measurement and outcomes used, the inclusion of broader economic impact was found to improve the attractiveness of vaccination. Further research is needed on how different tools and techniques can be used in combination to capture the broader impact of vaccination in a way that is consistent with other health economic evaluations. In addition, more country level evidence is needed from low and middle income countries to justify future investments in vaccines and immunization programs. Finally, the proposed broader economic impact framework may contribute towards better communication of the economic arguments surrounding vaccine uptake, leading to investments in immunization by stakeholders outside of the traditional health care sector such as ministries of finance and national treasuries.

## Background

In the last two decades, several vaccines have been developed that target a range of infectious diseases of global public health importance [1]. However, the list price of these vaccines in high income countries is substantially greater than for traditional vaccines [2]. Hence governments and external funders with limited resources often have to trade off purchasing vaccines against investing in other preventative and therapeutic interventions. In many high income countries, assessment of the relative cost-effectiveness of competing strategies is a key element to the adoption of new health technologies [3], alongside other considerations such as effectiveness, disease burden and equity impact. Such analyses usually focus on the health sector impact of vaccination, weighing the cost of an immunization program against the morbidity and mortality prevented, as well as potential savings derived from a reduction in health care utilization.

However, it has been suggested that these cost effectiveness analyses may present too narrow a perspective of the benefits of vaccination [4,5] and other child health programs [6]. This is particularly relevant to many low and middle income countries (LMICs), where decisions about health care spending are often made in the context of wider development goals, and not on issues restricted to the health care sector alone [7]. Furthermore, many such countries do not have locally established decision rules to facilitate the optimal allocation of resources within a fixed health care budget [8], so wider considerations such as the impact of immunization on economic growth or national budgets are often highly relevant.

Aside from their effectiveness in reducing disease and mortality, the economic benefits of vaccination have usually been measured in terms of the averted costs of medical care. Sometimes consideration is also made of the immediate productivity loss to patients (as a result of illness or death) and their carers. However, in the longer term, it has been suggested that vaccines can increase lifetime productivity due to better physical health, decreased chance of cognitive impairment caused by some vaccine preventable diseases, and better educational outcomes through increased school attendance [4,9]. It has also been suggested that reduced childhood mortality may encourage mothers to decrease the number of planned births, hence increasing household investment per child and enabling greater labor force participation [4,10]. In the long run, decreasing fertility may improve the dependency ratio and increase investment in physical and human capital [11,12].

On a community level, vaccination is associated with positive externalities due to its effect on the pathogen-host ecology [2,13]. For instance, vaccination can decrease the infection risk of unvaccinated people since they will no longer be at risk of becoming infected by vaccinated people (herd immunity). The use of vaccines may also reduce the need for antimicrobial use against affected organisms, hence reducing antimicrobial resistance. Vaccination programs may also improve the financial sustainability and affordability of healthcare programs in LMICs. Their use as part of a treatment cluster, or in combination with other infrastructure projects (such as water management systems) to maximize community health outcomes, offers opportunities for cost sharing between programs. Additionally, introduction of vaccination to disease endemic areas may boost private demand for vaccines within communities, which could enable partial cost recovery by health departments [14-22].

Traditional health economic evaluations only consider the sector-specific impact of interventions on the health care sector. However, large outbreaks of diseases such as cholera or pandemic influenza can affect other sectors of the economy such as manufacturing, tourism and transport [2,9,23,24]. In countries with a high disease burden, vaccination may modulate such outbreaks, and have a substantial impact on demand, supply, trade and investment in the wider economy.

Recently, several frameworks have been proposed by which these wider benefits of vaccination can be categorised [4,9]*.* However, the extent to which these broader benefits are considered in current economic evaluations of vaccines is unclear. This systematic review aims to identify the extent to which broader economic benefits are already incorporated within existing economic evaluations of vaccines in LMICs, and to examine the validity of the underlying methodologies and associated outcomes employed in estimating their effect. Through the use of a narrative approach, we investigate how inclusion of these broader effects could impact on the performance of healthcare programs, and also explore the potential for incorporating them within metrics which can be adequately interpreted by decision makers looking to compare returns on investment (ROI) against those in other sectors.

## Methods

A systematic review was conducted to identify economic evaluations capturing the benefits of vaccination apart from (i) health effects (morbidity and mortality averted), (ii) health treatment costs saved and (iii) short term productivity losses due to being ill or caring for someone ill. For the purpose of this review, “narrow benefits” are defined as health effects, healthcare costs and short-term productivity losses to patients and caregivers. These benefits are typically incorporated into traditional economic evaluations. They are generally short-term (lasting not much longer than the duration of the illness and its sequelae) and are restricted to the vaccinated individual and closely related individuals (such as caregivers). “Broader” benefits are defined as potential benefits of vaccination aside from these; they typically involve longer term effects and/or wider externalities other than individuals vaccinated and their caregivers.

The framework proposed in Bärnighausen *et al.*[4] also differentiates between narrow and broad benefits on these grounds. However, following our review we expanded Bärnighausen’s framework to encompass other externalities and macroeconomic-level benefits proposed in recent literature [9]. In our classification, benefit categories were grouped under three broad headings depending on whether they related to health gains (narrow), productivity gains or community externalities (see Table 1). Productivity gains related to outcomes were divided into narrow short-term outcomes (immediate disease or death) and long-term outcomes (effects of better health leading to improved physical, educational and cognitive outcomes). 

**Table 1:** Categorized list of the benefits of vaccination

**Category**	**Definition**	**Examples of outcomes**
*A. Health-related benefits*		
A1. Health gains	Reduction in morbidity and mortality	Cases averted
		Deaths averted
		Disability-adjusted life years saved
A2. Health care savings	Reduction in cost of health care borne by the public sector or private individuals	Costs saved
*B. Productivity-related benefits*		
B1. Productivity gains related to care	Reduction in lost days of work due to sickness or caring for a sick patient	Value of productivity gained
B2. Productivity gains related to short term outcomes	Reduction in lost days of work due to sickness or death of sick patient	Value of productivity gained
		Lifetime earnings
B3. Productivity gains related to long term outcomes	Increased lifetime productivity because better health improves cognition, educational attainment and physical strength	Educational outcomes
		Cognitive outcomes
		Lifetime earnings
B4. Productivity gains related to household behaviour	Economic improvements due to changes in household choices such as fertility and consumption/saving as a result of improved child health and survival	Productivity
		Female labour participation
		Household investment per child
		Dependency ratio
*C. Community externalities*		
C1. Ecological effects	Health improvements in unvaccinated community members as a result of ecological effects such as herd immunity and reduced antibiotic usage.	Indirect vaccine protection
		Prevalence of antibiotic resistance
C2. Equity	More equal distribution of health outcomes	Distribution of health outcomes
C3. Financial sustainability	Improved financial sustainability of health care programs as a result of synergies with vaccination programs and/or stimulation of private demand.	Financial benefits
		Private demand estimates
C4. Macroeconomic impact	Changes in the national economy or individual sectors of the economy.	Gross domestic product
		Production by economic sector

Definitions in categories A, B and C1 are adapted from Bärnighausen *et al.*[4].

We reviewed MEDLINE, EconLit and the National Health Service Economic Evaluation Database (NHS-EED) for English language articles published between January 1990 and July 2011. Search filters for identifying papers specific to vaccination, economic outcomes and LMICs were then applied in combination. In addition, specific keywords associated with the various benefit categories were also included in the search in combination with these filters. A separate search was conducted for the broader benefits identified by Bärnighausen *et al.*[4] (category B3-4 and C1-4 in Table 1), using category specific keywords. Full search terms can be found in the additional file [see Additional file 1. Grey literature reports and working papers published online were also identified through discussions with subject experts involved in a World Health Organization consultation on the broader economic impact of vaccination (Toronto, 2011). To ensure reproducibility of search results, grey literature reports not publicly available on the internet were excluded. Reference lists in identified articles were scanned to identify additional studies missed during the search (snowball method).

Each study identified through the database search was initially categorized on the basis of its title and abstract to determine its possible relevance to the review. Studies could be economic evaluations, reviews, epidemiological studies (observational or experimental), contingent valuation studies or descriptions of measurement tools. Articles were then read to ensure that they met the following criteria: (i) in humans, (ii) not studies of travel vaccines, (iii) discussed or measured at least one of the benefits of vaccination listed in categories B3-4 or C1-4 of Table 1, (iv) conducted in LMICs, (v) discussed both cost and benefits of vaccines, (vi) discussed economic consequences of vaccine use (and not purely epidemiological studies looking at the impact of vaccination on infection or disease).

## Results

### Overview

Full texts of 92 articles were obtained. Of these, 26 were found to meet the selection criteria, and were included in the qualitative synthesis. Three of these 26 studies were grey literature reports or working papers and were not retrieved from a database [25-27]. A PRISMA flow diagram of search results [see Additional file 2, and a detailed description of all included studies [see Additional file 3 can be found as additional files. Half the studies incorporated both narrow and broad economic benefits of vaccination, while the other half solely incorporated broader benefits. Perhaps reflective of growing interest in the measurement of broader benefits, only three included studies were published in 2005 or earlier [11,14,28]. Five studies also estimated economic gains from vaccine use in multiple LMICs [11,22,29-31].

Table 2 shows the number of articles mentioning different categories of benefit, and the associated vaccine antigens and targeted age groups discussed. While there were no clear patterns, on the whole outcome-related productivity gains and equity were discussed most often in articles dealing with vaccines targeted at children under five years old. Financial sustainability was discussed most often in articles dealing with vaccines targeted at both children and adults. Ecological externalities were discussed equally often in both types of articles. Also, the majority of the studies using a willingness-to-pay approach to estimate societal value of a vaccine were in articles dealing with older age groups (Table 1).

**Table 2:** Number of articles by vaccine antigen, target age range and category of broader benefit mentioned

	**Outcome- related productivity**	**Financial sustainability**	**Ecological externalities**	**Equity**
**Children only (under five years)**				
Diptheria, tetanus, pertussis				1
Haemophilus influenzae type B	3	1		
Hepatitis A	3		1	
Hepatitis B				
Malaria	1			
Measles	3			2
Meningitis	5			
Pertusiss	1			
Pneumococcus	2	1	3	
Polio	3	1		1
Rotavirus	4			
Tuberculosis	3			1
Yellow fever	2			
**Children and adults**				
Cholera		4	4	
Dengue		1		
Malaria		2		
HIV	1	1		
Typhoid		1		
Total*	32	12	8	5

* Totals add up to more than 26 because some articles discuss multiple vaccine antigens.

### Productivity gains related to long-term outcomes (B3)

Productivity gains related to long-term outcomes were included in eight studies [11,22,25-27,30-32]. Three were primary studies (one cluster randomised trial [25] and two observational longitudinal studies [26,32]) examining the long term productivity effects of vaccination against childhood diseases. Barham and Calimeris [25] used a quasi-randomised cluster design to find that early childhood vaccination combined with a family planning program in Bangladesh significantly improved cognitive outcomes in later childhood. The effect was equivalent to completing three years of primary school. Bloom *et al.*[32] found that children in the Philippines who received a package of six vaccines by age two years had significantly better scores on cognitive tests at 10–11 years old. However, Kumar [26] found mixed evidence in India. Children receiving vaccines in the universal immunization program had lower primary school completion rates, but higher secondary school completion rates. Kumar suggested that the disappointing primary school completion results could be due to poorer health among the children who would not have survived without vaccination, or lower household investment in children due to their unexpected survival.

Five studies explored techniques for converting gains in cognitive ability into measurable economic benefits that can be used in economic evaluations. Connolly and Constenla [27] examined the effect that vaccination may have on net tax revenues, via productivity gains as a result of changes to health and educational outcomes. Hence reduced disease could result in improved education, and a subsequent increase in tax revenue later in life. Bloom *et al.*[11] used the link between health and wages [10] to project the long-term economic benefit of improved childhood health. Tebbens *et al.*[30] projected the lifetime impact of averting paralysis due to polio in a cost-effectiveness analysis of the global polio eradication initiative. Stack *et al.*[31] estimated the economic impact of vaccines in 72 countries eligible for assistance through the GAVI Alliance. This incorporated both narrow benefits (health care costs) as well as productivity loss due to both long-term disability and premature mortality. An extension of this study also estimated the value of deaths averted using the value of statistical life approach, which measures willingness to pay to avert such deaths [22].

### Ecological externalities (C1)

Vaccination can reduce disease incidence in unvaccinated individuals by preventing infection transmission from vaccinated to unvaccinated individuals. This positive externality is called indirect or “herd” protection. The size of the externality is a nonlinear function of the proportion of the population vaccinated [33]. Ecological externalities were captured in five cost-effectiveness analyses [34-38] and three cost-benefit analyses [20,39,40]. One of the cost-benefit analyses was based on willingness-to-pay values from a contingent valuation study [20]. Of these, six captured the effect of ecological externalities in the base case [20,34,35,37,39,40] and two in sensitivity analyses [36,38]. All the studies found that including herd immunity resulted in more favorable cost-effectiveness or cost-benefit ratios and an increase in the predicted number of cases averted by vaccination.

Giglio *et al.*[37] opted for the simplest (and most approximate) approach, assuming that the number of indirectly prevented cases is just a multiple of the number of directly vaccinated individuals. Kim *et al.*[36] and Vespa *et al.*[38] examined the cost-effectiveness of different pneumococcal conjugate vaccines in the Gambia and Brazil respectively. They accounted for herd protection (as well as serotype replacement) by constructing various scenarios about the magnitude of this effect based on the post-vaccination experience of other countries such as the United States. However, the most complete approach was to incorporate herd protection using epidemiological models that represent infection transmission between infected and susceptible individuals. Jeuland *et al.*[35,39,40] and Cook *et al.*[20] conducted cost-effectiveness and cost-benefit evaluations of cholera vaccines in several LMICs. Herd protection was assumed to be a function of vaccine coverage using results from epidemiological models by Longini *et al.*[41]. They found that herd protection effects were a key factor in the different economic attractiveness of school-based versus community-based vaccination programes. Lopez *et al.*[34] developed a *de novo* transmission dynamic model which could capture the indirect benefit of vaccination to examine the cost-effectiveness of hepatitis A vaccination in Argentina.

### Equity (C2)

Cost-effectiveness analysis usually considers an improvement in health to be of equal value regardless of the person whose health is improved. However, public investment in vaccination may change the distribution of health in a population. Two studies have suggested that the greatest gains may be in sections of the population who are disadvantaged in terms of their existing health or their socioeconomic status, thus reducing health inequalities [28,42]. A study of measles vaccination in Bangladesh found that unvaccinated children in the poorest quintile were more than twice as likely to die as those from the least poor quintile, and that vaccination reduced socioeconomic status- related mortality differentials [28]. Similarly, a study of delivering a package of five vaccines in Ghana found that children from poorer households benefit more from immunization than those from relatively better-off households [42].

### Sustainability (C3)

Immunization programs provide opportunities for cost-sharing and external funding with other health and social interventions, through their ability to be included as part of combined treatment packages, development projects or social initiatives like poverty reduction campaigns. Three evaluations estimated the cost-effectiveness of vaccination when used alongside another public health intervention [29,30,40]. Jeuland and Whittington [40] found a greater percentage of their model scenarios to have a favorable cost-benefit ratio for combination strategies of vaccination and water supply improvements versus just vaccination. Additionally, the cost-benefit ratio varied depending on the order sequence of water pump installation and vaccination initiation. Similarly, Niessen *et al.*[29] investigated the cost-effectiveness of multiple interventions (including vaccination) against pneumonia, and found that different combinations of expanding vaccine coverage with community or facility-based case management, nutritional programs, or indoor air pollution measures maximized child health by providing the greatest health yield per dollar spent. Such studies are especially useful for conditions such as pneumonia in which non-bacterial causes can be major contributors towards disease burden.

Vaccination programs may also improve health care infrastructure and supply chains that could also be used for other public health initiatives. Tebbens *et al.*[30] found that polio eradication programs had previously unaccounted for net benefits associated with vitamin A supplement delivery.

Immunization programs can result in increased demand for vaccines. When the cost of vaccine purchase is subsidized, some members of the population may be able and willing to pay out-of-pocket to be vaccinated, which would reduce the financial burden on the healthcare system. Nine of the reviewed studies estimated private demand for vaccination using a willingness-to-pay approach [14-22]. These suggested that unvaccinated individuals could be protected at no extra cost if the government set a user fee that was below the average willingness-to-pay for a particular vaccine. In certain situations, it may even be possible to set user charges at levels sufficient for partial cost recovery without loss of demand. Interestingly, several of the studies also incorporated the social benefit of herd immunity in their assessments [20,39].

Hence, generation of private demand could improve the affordability and financial sustainability of immunization programs. However, issues such as ensuring that vaccines are available to members of the population unable to pay user charges and avoiding free riders who rely on indirect protection only will need to be considered.

## Discussion

This review shows that several broader categories of economic benefits associated with vaccination in LMICs are being captured in primary studies and quantified in economic evaluations. Our search identified 26 studies which assessed at least one broader benefit of an immunization program.

The first category involves productivity gains because of reduced disease risk. Of these, productivity gains related to care and to short-term outcomes are more commonly captured in economic evaluations. Productivity gains related to long-term outcomes and to household behavior are not commonly estimated or incorporated in economic evaluations [4]. There may be a number of reasons for this. Firstly, evidence for many of the proposed benefits of vaccination is still limited. Vaccine trials do not routinely incorporate cognitive, educational and behavioral outcomes. Furthermore, the final endpoint in such an analysis is the productivity of an adult of working age who either was or was not vaccinated as a child. Such an endpoint may occur too long after the vaccination event to be feasibly collected. There are also ethical problems in keeping trial participants unvaccinated once a vaccine has been shown to be safe and effective. Hence existing evidence is largely based around retrospective observational cohorts, which may be biased since they rely on controls with unvaccinated individuals.

Secondly, the economic modeling framework within which such benefits can be incorporated is still unclear. While many of the reviewed studies were able to quantify the productivity-related benefits of vaccination, apart from care-related and short-term outcome-related productivity effects this was not routinely incorporated into summary statistics such as cost-effectiveness or cost-benefit ratios. The most common means of incorporating productivity gains was the human capital approach, which values the remaining years of productive employment a person can have up to retirement based on national average incomes [43]. However, this method only captures productivity loss due to death or disability, and not due to cognitive or educational deficits as a result of childhood illness. Furthermore, the method has been criticized in its own right, since it assumes that the work a sick or deceased worker does cannot be replaced by someone currently unemployed or by simply reassigning it to other workers [44]. Possibly for this reason, lost income as a result of premature death was rarely incorporated in the reviewed studies, even when economic evaluations used the human capital approach to value productivity loss. Other methods (such as the friction cost approach) are less likely to overestimate productivity loss due to illness, but are equally difficult to use to capture the effects of long-term outcomes [44].

None of the studies accounted for behavior related productivity gains such as potential reductions in fertility as a result of improved child survival. In principle, lifetime benefit models (such as by Connolly and Constenla [27] and Bloom *et al.*[11]) are able to quantify the effect of demographic changes such as dependency ratio improvements or productivity changes from increased female workforce participation. However, primary studies are still needed to explore the link between vaccination and household decisions such as childbearing and investment spending.

Value of statistical life methodology, used by Ozawa *et al.*[22], offers a different approach by valuing lives based on individuals’ willingness to pay to reduce the risk of death rather than the economic output accrued to a year of life. This method often produces estimates of monetary value of prevented deaths that are far greater than those estimated using other methods (see for example Molinari *et al.*[45]). While this does not mean it is wrong, it does raise issues of comparability with existing economic evaluations and burden estimates conducted using more conservative methods. Another challenge in applying this methodology on a global level is the difficulty in knowing the extent to which to apply normative values on life (which mostly originate from high income countries) across national borders. Traditional cost-effectiveness analyses sidestep this problem by valuing health equally across the world in non-monetary terms; differences in decision making across countries hence stem from differences in thresholds representing societal willingness to pay for health (which reflect differences in budgets and national priorities) and in the direct economic impact of disease.

Ecological externalities, particularly herd immunity, were the most common broader benefit to be incorporated into economic evaluations. This is likely to be because the techniques for such analyses (such as the use of “transmission dynamic models”) are already well-established in the epidemiological and health economics fields [13]. However, many LMICs lack the capacity and/or surveillance systems to perform and parameterize these sophisticated analyses [46]. For example, infection transmission models capturing the indirect effects of vaccination may require data on behavioral and contact patterns which are not readily available in many LMICs. Besides herd immunity, negative ecological externalities may also occur such as replacement of strains of pathogens eliminated by vaccination with other strains unaffected by vaccination [13].

Equity, affordability and financial sustainability are often important considerations for vaccine introduction in LMICs. However, trials do not to routinely collect economic information by socioeconomic strata. Such data would enable analyses of the impact of vaccines on equity or financial sustainability of other interventions, as well as elucidate any methodological difficulties with incorporating them into standard cost-effectiveness frameworks. This is evident in the case of certain spill over effects of immunization programs. Reuse of immunization infrastructure, better surveillance, human resource gains, and improved drug procurement systems, are some potential benefits of immunization programs which are rarely incorporated into cost effectiveness studies [47]. Other frameworks such as return on investment analyses or optimization modeling may be able to address some of these issues more effectively, and provide more comprehensive representation of the societal value of vaccination.

None of the identified studies took a macroeconomic approach to evaluating the impact of vaccination, as proposed by a recent World Health Organization guidance document [9]. Such an approach has been used to evaluate the impact of vaccination during an influenza pandemic in several European countries, using computable general equilibrium models [48], but an LMIC application or an application to situations beyond pandemics (such as an endemic disease) has yet to be published.

This review has limitations, because it was designed to give a broad qualitative overview of existing literature rather than to enable detailed quantitative synthesis. Only one person was responsible for study selection, and the studies were not weighted by quality scores. However, the permissive inclusion criteria ensured that a variety of study types and measurement techniques were reviewed, including those employing unconventional tools and techniques. Also, our study was restricted to methods applied to LMICs only, since it was motivated by decision making in these settings, where stakeholders often require information not provided by conventional economic evaluation methods. As a result, it may not have captured novel approaches being developed or applied in high income settings.

Most (22/26) of the reviewed studies included only a single category of broader economic impact. There may be several reasons for this. Most (16/26) studies were observational, willingness to pay, return on investment or cost of illness studies rather than full economic evaluations (cost-effectiveness or cost-benefit analyses) attempting to capture all important costs and outcomes of an intervention. However, even the full economic evaluations presented a limited number of broader categories of impact, even though a comprehensive range of traditional (narrow) measures were presented, such as direct medical costs, cases avoided or lives saved. This may be due to the current novelty of these measures, as well as the lack of comprehensive guidelines for economic evaluations about which of these broader measures should be reported and in what way. A further difficulty is the complexity of the relationships between vaccination, health and broader economic outcomes, which require a range of types of evidence and techniques to quantify. Given the difficulty with both measurement and interpretation, it may be impractical to develop a single composite measure capturing all relevant economic benefits of vaccination. Instead, several evaluation techniques may need to be implemented to obtain a representative set of outcome measures. However, there is little guidance about the way several categories of benefits estimated using different techniques can be combined in the same evaluation.

## Conclusions

Broader categories of economic benefits such as those reviewed here offer valuable information to decision makers, especially those outside the health sector. However, further work on techniques to value such categories and combine them in economic evaluations is still needed.

Key recommendations arising from this review are summarized below:

Studies linking changes in health to long-term behavioral and developmental outcomes are needed, as well as techniques to analyze observational studies in ways that reduce potential biases.

Information needs of stakeholders from different sectors (including health, finance and external donors) should be obtained to guide incorporation of broader benefits into economic evaluation, as well as their effective communication.

Guidelines from decision makers and agencies setting standards for economic evaluations are needed about appropriate ways to incorporate broader economic benefits, particularly about combining several benefit categories or evaluation techniques.

Pilot testing and validations studies of evaluations based on such normative guidelines should be conducted in LMICs.

## Competing interests

The authors declare that they have no competing interests.

## Authors’ contributions

MJ, RH and RD conceived the study. The search filters were developed by RD and reviewed by MJ. Initial categorisation of search results based on title and abstract and the subsequent review of articles for inclusion was conducted by RD. The final list of included articles was reviewed by MJ and RH. RD reviewed the shortlisted articles with input from MJ, RH, IvdP and SE. RD summarised the literature and wrote the first draft of the manuscript with input from MJ and RH. All authors contributed to the writing of the manuscript. All authors read and approved the final manuscript.

## Pre-publication history

The pre-publication history for this paper can be accessed here:

http://www.biomedcentral.com/1471-2458/12/878/prepub

## Supplementary Material

Additional file 1 Search terms used in the systematic review. Click here for file

Additional file 2 Flow diagram of search results. Click here for file

Additional file 3 Summary tables of all included studies. Click here for file
