# Supplementary material for: Systematic review of studies evaluating the broader economic impact of vaccination in low and middle income countries
Source: BMC Public Health. 2012 Oct 16;12:878. doi: 10.1186/1471-2458-12-878 (PMC3532196; doi:10.1186/1471-2458-12-878)
Supplement: Additional file 1 — Search terms used in the systematic review. [file 1471-2458-12-878-S1.docx]

Additional file 1:

Category specific search terms used in database search

| **Category** | **Keywords used** |
| --- | --- |
| B3 - Productivity gains related to long term outcomes | lifetime productivity, earning$, income, labour, sick days, cognition, cognitive ability, long term disability, mental ability, skills development, school attendance, education, educational outcomes, workforce, GDP |
| B4 - Productivity gains related to household behaviour | fertility, pregnancy, child mortality, female workforce, female employment, labour participation, dependency ratio |
| C1 - Ecological effects | herd effects, herd immunity, antibiotic resistance, drug resistant strain, transmission dynamic model, serotype replacement, non-vaccine serotype |
| C2 - Equity | equity, horizontal equity, vertical equity, distribution$ |
| C3 - Financial sustainability | resource sharing, treatment cluster, cost splitting, coverage, uptake |
| C4 - Macroeconomic impact | GDP, national income, foreign investment, consumption, saving$, output, growth, sectoral loss |
