# Supplementary material for: Systematic review of studies evaluating the broader economic impact of vaccination in low and middle income countries
Source: BMC Public Health. 2012 Oct 16;12:878. doi: 10.1186/1471-2458-12-878 (PMC3532196; doi:10.1186/1471-2458-12-878)
Supplement: Additional file 2 — Flow diagram of search results. [file 1471-2458-12-878-S2.docx]

Additional file 2:

Flow diagram of search results

Additional records identified through other sources
(n = 3 )

Records identified through database searching
(n = 1107 )

Studies included in qualitative synthesis
(n = 26 )

Full-text articles excluded, with reasons
(n = 66 )

Full-text articles assessed for eligibility
(n = 92 )

Records excluded
(n = 575 )

Records screened
(n = 667 )

Records after duplicates removed
(n = 667 )

## Identification

## Eligibility

## Included

## Screening
