# Supplementary material for: Systematic review of studies evaluating the broader economic impact of vaccination in low and middle income countries
Source: BMC Public Health. 2012 Oct 16;12:878. doi: 10.1186/1471-2458-12-878 (PMC3532196; doi:10.1186/1471-2458-12-878)
Supplement: Additional file 3 — Summary tables of all included studies. [file 1471-2458-12-878-S3.docx]

Additional file 3:

Summary table for all included studies.

| ***Year*** | ***Author*** | ***Vaccine/Country*** | ***Study type*** | ***Traditional outcome(s)*** | ***Broader economic impact(s) included*** |
| --- | --- | --- | --- | --- | --- |
| 2003 | Bishai *et al* [25] | Measles/ Bangladesh | Longitudinal | N/A | *C2 :* Health equity – under 5 mortality rate |
| 2005 | Bloom *et al* [11] | Multiple^/ 72 LMIC | ROI | Deaths avoided | *B3:* Annual earnings increase (survival to adult age) |
| 2005 | Sauerborn *et al* [14] | Malaria/ Burkina Faso | WTP | N/A | *C3:* Private demand |
| 2007 | Lopez *et al* [34] | Hepatitis A/ Argentina | CEA | Cases & deaths avoided. Life years averted. | *C1:* Herd effect |
| 2007 | Lucas *et al* [15] | Cholera/ Mozambique | WTP | N/A | C3: Private demand |
| 2008 | Barham & Calimeris [29] | Measles, DPT, Polio/ Bangladesh | Longitudinal | N/A | *B3:* Cognitive test performance |
| 2008 | Kim *et al* [17] | Cholera/ Vietnam | WTP | N/A | *C3:* Private demand |
| 2008 | Palanca-Tan [16] | Dengue/ Phillipines | WTP | N/A | *C3:* Private demand |
| 2008 | Whittington *et al* [18] | HIV/ Thailand | WTP | N/A | *C3:* Private demand |
| 2009 | Connolly & Constenla [32] | Malaria/ Ghana | ROI | Direct medical costs, caretaker productivity loss | *B3:* Lost earnings (sickness & death), increased earnings due to better educational outcomes |
| 2009 | Cook *et al* [20] | Cholera/ India | WTP | Cost of illness including caretaker productivity loss | *C1:* Herd effect;  *C3:* Private demand |
| 2009 | Jeuland & Whittington [40] | Cholera/  N/A | CBA | Cases & deaths avoided, COI avoided | *C1:* Herd effect;  *C3:* Intervention mixes |
| 2009 | Jeuland *et al* [39] | Cholera/ Mozambique | CBA | Cases prevented | *C1:* Herd effect |
| 2009 | Jeuland *et al* [35] | Cholera/ Bangladesh, India, Indonesia, Mozambique | CEA | Cases, deaths avoided, DALYs averted, public COI avoided | *C1:* Herd effect |
| 2009 | Kumar [31] | BCG, Polio, DPT, Measles/ India | Longitudinal | N/A | *B3:* School completion rates |
| 2009 | Niessen *et al* [26] | Pneumonia/ 40 LMIC | CEA | DALYs averted | *C3:* Intervention mixes |
| 2009 | Whittington *et al* [19] | Cholera, Typhoid/ India | WTP | N/A | *C3:* Private demand |
| 2009 | Vespa *et al* [38] | Pneumococcal disease/ Brazil | CEA | Cases & deaths avoided, DALYs averted, COI avoided, caretaker productivity loss | *C1:* Herd effect |
| 2010 | Bawah *et al* [42] | BCG, Polio, DPT, Measles/ Ghana | Longitudinal | N/A | *C2:* Health equity – under 5 mortality rate/ survival odds |
| 2010 | Giglio *et al* [37] | Pneumococcal disease/ Argentina | CEA | Cases & deaths avoided, life years gained, COI avoided, caretaker productivity loss | *C1:* Herd effect |
| 2010 | Kim *et al* [36] | Pneumococcal disease/ Gambia | CEA | Cases & deaths avoided, DALYs averted, caretaker productivity loss. | *C1:* Herd effect, serotype replacement |
| 2010 | Udezi *et al* [21] | Malaria/ Nigeria | WTP | N/A | *C3:* Private demand |
| 2011 | Bloom et al [30] | BCG, Polio, DPT, Measles/ Philippines | Longitudinal | N/A | *B3:* Cognitive test performance |
| 2011 | Ozawa *et al* [22] | Multiple*/ 72 LMIC | Cost of Illness/ WTP | N/A | *B3:* Value of statistical life;  *C3:* Private demand |
| 2011 | Stack *et al* [28] | Multiple*/ 72 LMIC | Cost of Illness | Cases & deaths avoided, COI avoided, caretaker productivity loss | *B3:* Lost productivity (sequelae & premature death) |
| 2011 | Tebbens *et al* [27] | Polio/ 104 LMIC | CEA & CBA | Cases & deaths avoided, DALYs averted | *B3:* Lost productivity (paralysis);  *C3:* Intervention mixes |

^ Haemophilus influenzae type B, Hepatitis B, yellow fever, rotavirus, pneumococcal disease, meningitis

*Pertussis, Measles, Rotavirus, Haemophilus influenzae type B pneumonia, pneumococcal pneumonia, meningitis, malaria
